# A Handheld Multispectral Device for Assessing Leaf Nitrogen Concentrations in Maize

**DOI:** 10.3390/s25133929

**Published:** 2025-06-24

**Authors:** Felipe Hermínio Meireles Nogueira, Adunias dos Santos Teixeira, Sharon Gomes Ribeiro, Luís Clênio Jario Moreira, Odílio Coimbra da Rocha Neto, Fernando Bezerra Lopes, Ricardo Emílio Ferreira Quevedo Nogueira

**Affiliations:** 1 Department of Agricultural Engineering, Federal University of Ceara, Fortaleza 60455-760, Brazil; adunias@ufc.br (A.d.S.T.); odilioneto@gmail.com (O.C.d.R.N.); lopesfb@ufc.br (F.B.L.); 2 Soil Science Graduate Program, Federal University of Ceara, Fortaleza 60455-760, Brazil; 3 Department of Agronomy, Federal Institute of Education, Science and Technology of Ceara, Limoeiro do Norte 62930-000, Brazil; cleniojario@gmail.com; 4 Department of Metallurgical and Materials Engineering, Federal University of Ceara, Fortaleza 60455-760, Brazil; emilio@ufc.br

**Keywords:** spectroradiometry, precision agriculture, technological development, *Zea mays* L.

## Abstract

**Highlights:**

**What are the main findings?**

- A handheld multispectral device using the AS7265x sensor was successfully developed and validated.
- The use of multivariate statistical models (PLSR and PCR) significantly enhanced the accuracy of predicting leaf nitrogen concentration in maize.
- The device enabled rapid, non-destructive nitrogen estimation under real field conditions.

**What is the implication of the main finding?**

- The successful development and validation of the handheld device demonstrates the feasibility of integrating portable multispectral sensors into precision agriculture for effective on-site nutrient assessment.
- The improved prediction accuracy provided by the multivariate statistical models supports reliable nitrogen estimations, which can lead to more optimized nutrient management decisions.
- The device’s ability to conduct rapid, non-destructive measurements under field conditions facilitates timely interventions and supports sustainable agricultural practices.

**Abstract:**

This study presents the MSPAT (Multispectral Soil Plant Analysis Tool), a device designed for assessing leaf nitrogen concentrations in maize crops under field conditions. The MSPAT includes the AS7265x sensor, which has 18 bands and covers the spectrum from 410 to 940 nm. This device was designed to be portable, using the ESP32 microcontroller and incorporating such functionalities as data storage on a MicroSD card, communication with a smartphone via Wi-Fi, and geolocation of acquired data. The MSPAT was evaluated in an experiment conducted at the Federal University of Ceará (UFC), where maize was subjected to different doses of nitrogen fertiliser (0, 60, 90, 120, 150, and 180 kg·ha^−1^ N). Spectral readings were taken at three phenological stages (V5, V10, and R2) using the MSPAT, an SPAD-502 chlorophyll meter, and a FieldSpec PRO FR3 spectroradiometer. After the optical measurements were taken, the nitrogen concentrations in the leaves were determined in a laboratory by using the Kjeldahl method. The data analysis included the calculation of normalised ratio indices (NRIs) using linear regression and the application of multivariate statistical methods (PLSR and PCR) for predicting leaf nitrogen concentrations (LNCs). The best performance for the MSPAT index (NRI) was obtained using the 900 nm and the 560 nm bands (R^2^ = 0.64) at stage V10. In the validation analysis, the MSPAT presented an R^2^ of 0.79, showing performance superior to that of SPAD-502, which achieved an R^2^ of 0.70. This confirms the greater potential of the MSPAT compared to commercial equipment and makes it possible to obtain results similar to those obtained using the reference spectroradiometer. The PLSR model with data from the FieldSpec 3 provided important validation metrics when using reflectance data with first-derivative transformation (R^2^ = 0.88, RMSE = 1.94 and MAE = 1.28). When using the MSPAT, PLSR (R^2^ = 0.75, RMSE = 2.77 and MAE = 2.26) exhibited values of metrics similar to those for PCR (R^2^ = 0.75, RMSE = 2.78 and MAE = 2.26). This study validates the use of MSPAT as an effective tool for monitoring the nutritional status of maize to optimize the use of nitrogen fertilisers.

## 1. Introduction

Precision agriculture (PA) has emerged as a key approach to promoting sustainability in agricultural production systems. Of note among these systems is variable rate fertilisation, which adjusts fertiliser application based on the specific characteristics of the soil and the nutritional needs of crops. This method makes it possible to optimise the use of fertilisers, achieve high yields, and reduce impacts on the environment. PA also involves pest and disease management, allowing the targeted application of pesticides to reduce the use of chemicals and minimise environmental contamination. In this context, proximal sensing technology is considered an efficient alternative for acquiring real-time spectral data directly from agricultural targets, enabling rapid and non-destructive assessments of plant status [1,2].

Recent advances in electronics, including improvements in optical sensors, microcontrollers, and data-processing techniques, have significantly enhanced the effectiveness and applications of proximal sensing in precision agriculture [3,4]. The use of multispectral sensors provides information about the spectral behaviour of targets and allows the estimation of nitrogen concentrations in plants [5]. For this reason, an AS7265x sensor (SparkFun Electronics) has demonstrated high potential for analysing the health of agricultural crops [6,7].

Given the diversity of available technologies, using sensors or instruments for collecting spectral data requires specific expertise in fields such as optics, electronics, and data processing. Additionally, proper application requires specialized tools, including calibration standards, signal-processing software, and data-modelling approaches tailored to agricultural conditions. Cilia et al. [8] and Ma et al. [9] have used hyperspectral UAV data to assess wheat grain protein and optimize variable-rate fertilisation, while Peng et al. [10] developed an in situ spectral method for quantifying nitrogen, phosphorus, and potassium levels across multiple plant species. These applications demonstrate how different tools and methodologies must be carefully selected based on the complexity of spectral analysis and the target parameters.

Nitrogen (N) is one of the most important nutrients required by plants, as it is an essential component of proteins, nucleic acids, and chlorophyll. Its availability directly influences plant growth, photosynthesis, and metabolic functions, making efficient management crucial to maintaining productivity and minimising environmental impacts [11]. Berger et al. [12] presented the state-of-the-art in nitrogen (N) monitoring of agricultural crops, highlighted important aspects for exploring the spectrum of leaves, and suggested new approaches to use bands in the infrared spectrum. The focus on this spectral region is due to the high nitrogen content in proteins, which exhibit absorption peaks primarily in the SWIR (short-wave infrared) region, particularly at 2180 and 2300 nm [13,14]. Spectroscopy has been widely used to estimate leaf nitrogen concentrations, particularly through the analysis of specific spectral regions. Min and Lee [15] highlighted the green and red-edge spectral bands (546 and 722 nm) as being significant for nitrogen estimation in citrus crops, achieving correlation coefficients greater than 0.5 in the 553 and 707 nm bands. These findings reinforce the potential of remote sensing as an effective tool for assessing plant nutrition, enabling rapid and non-destructive estimation of the nutritional statuses of plants.

Statistical and programming tools are integrated into spectral analysis techniques to identify the most relevant bands for agricultural applications and establish reliable correlations between spectral reflectance and plants’ biochemical compositions. Leaf nitrogen estimation relies on statistical modelling, where multivariate approaches such as partial least squares regression (PLSR) and principal component regression (PCR) help refine the analysis by minimising redundancy among sensor bands and improving the robustness of the estimates. These methods ensure that spectral responses provide valuable quantitative assessments of plant nutrient status, enabling more precise monitoring and optimisation of fertilisation strategies [16,17].

Developing a portable optical instrument for leaf tissue analysis in an agricultural environment requires multidisciplinary knowledge, encompassing electronics, programming, modelling, 3D printing, and materials engineering for sensor calibration. The development of a handheld multispectral device for the non-destructive assessment of leaf nitrogen concentrations (LNCs) in maize is crucial for advancing precision agriculture and improving real-time nutrient monitoring methods. Traditional approaches to evaluating LNC often rely on labour-intensive procedures or costly spectroradiometric instruments. The device presented here integrates an AS7265x sensor with ESP32-based communication, offering features such as data storage, Wi-Fi connectivity, and geolocation to streamline data collection and facilitate large-scale implementation. Additionally, the incorporation of a highly reflective calibration plate made of sintered barium sulphate ensures a cost-effective reliable sensor calibration strategy, maintaining accuracy in spectral measurements.

Thus, the main objective of this study is to present the development and application of a handheld tool for assessing leaf nitrogen concentrations in maize crops under field conditions.

## 2. Materials and Methods

### 2.1. Device Design and Construction

The multispectral optical device uses the AS7265x (SparkFun Electronics, Niwot, CO, USA) sensor for spectral characterisation of the target. This sensor allows a detailed evaluation of the optical characteristics of the target by integrating three photodiodes that cover a range of 18 bands (410 to 940 nm) with an FWHM (full width at half maximum) of 20 nm over the VNIR spectrum (visible and near-infrared). In addition, the sensor is small (4.7 × 4.5 mm) and affordable and can be easily integrated into microcontrollers, making it suitable for field applications. The sensor was integrated into the ESP32 (Espressif Systems, Shanghai, China) microprocessor due to its portability, high processing capacity, and ability to transmit data via Wi-Fi or Bluetooth. The GY-NEO6MV2 (u-blox AG, Thalwil, Switzerland) GPS module was included as an accessory to help analyse the spatial distribution of the spectral data. The device communicated with the operator via an OLED display, which provided information on the number of samples obtained, the accuracy of the GPS signal, and the power level of the battery. To ensure a longer operating time in the field, we used two 18650 Li-Ion batteries with a capacity of 2200 mAh connected to a TP4056 (NanJing Top Power ASIC Corp., Nanjing, China) charging module. The data obtained by the device are stored on a MicroSD card connected to a card reader, and they are available in CSV format. In addition, the Wi-Fi communication capacity of ESP32 allows data to be transmitted to an IP address that can be accessed by a smartphone browser.

**Figure 1:**
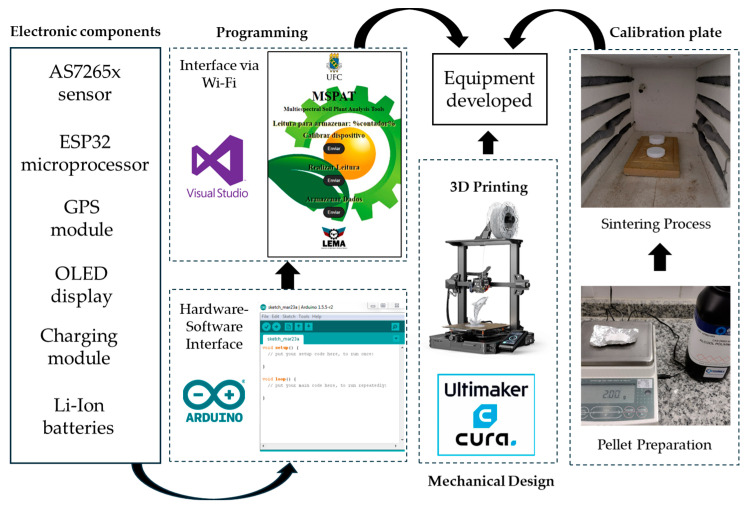
Flowchart of the stages of the equipment component creation process.

The parts made of PLA (polylactic acid) were 3D printed using UltiMaker Cura 3.6 software via an Ender-3 S1 printer (Creality, Shenzhen, China). The ESP32 microprocessor was programmed using the Arduino IDE 2.3.6 platform, and the Wi-Fi communication interface of the smartphone was developed using Visual Studio 2022 v17.14 software. These activities were carried out at the Laboratory of Electronics and Agricultural Mechanisation (LEMA) of the Federal University of Ceará (UFC). The flowchart presented in Figure 1 illustrates the stages in building the equipment components, encompassing programming, 3D printing, and the development of the reference plate used for sensor calibration.

### 2.2. Calibration of the Optical System

A reference standard was developed using highly purified sintered barium sulphate as an alternative to Spectralon, as the corresponding materials are easily accessible and the corresponding methodology is simple. This material was selected due to its high reflectance and diffuse light-scattering properties, which are essential for ensuring the accuracy of optical measurements. For reflectance sensors, it is crucial to use a calibration plate with high reflectance as a reference for converting raw sensor outputs into absolute reflectance values. The selected material has properties similar to a Lambertian surface, ensuring uniform reflectance distribution and consistent measurement conditions. In this manner, the calibration plate compensates for variations in the light source and sensor responsivity, thereby enhancing measurement precision by providing a reliable reference standard [18]. A reference board was constructed based on the methodology described by Poh et al. [19], using three pellets of sintered barium sulphate. The manufacturing stages took place at the Materials Characterisation Laboratory (LACAM) of the Department of Metallurgical and Materials Engineering and at the Pedology Laboratory of the Department of Soil Sciences at UFC.

### 2.3. Experimental Site and Design

**Figure 2:**
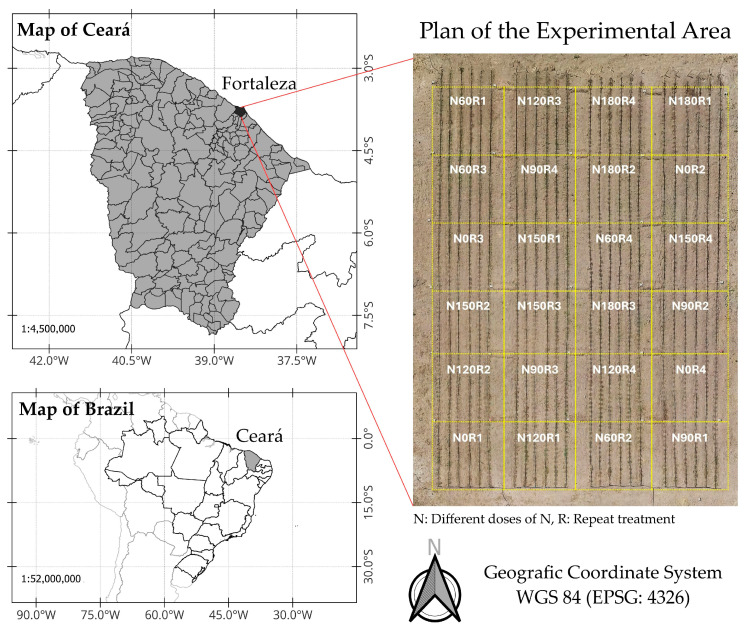
Map showing the location of the experimental area.

A field study involving the use of maize to calibrate the sensor was conducted in the experimental area of LEMA on the Pici Campus of the Federal University of Ceará in Fortaleza (Figure 2), located at latitude −3.745294 and longitude −38.581159 at an altitude of 19 m. According to the Köppen classification, the climate in the experimental area is of the Aw’ type. The soil in the area is classified as a red-yellow Argisol with a sandy clayey-loam texture. We study included the AG-1051 maize cultivar, recommended for silage and green maize production. To provide different field conditions for N in the leaf tissue, the maize crop, under drip irrigation, was subjected to different doses of nitrogen fertiliser (urea) in a completely randomised design (CRD). Six treatments, namely N0, N60, N90, N120, N150, and N180, corresponding to 0, 60, 90, 120, 150, and 180 kg·ha^−1^ N, respectively were applied with four replications, totaling 24 experimental units. Fertilisation was split according to the plan shown in Table 1. The other nutrients were apportioned such that the demands of the crop would be met and supplied based on the results of the soil analysis (Table 2) using the following fertilisers: potassium chloride, Single Super Phosphate, MAP (monoammonium phosphate), magnesium sulphate, and Microcomplex^®^ (Biolchim do Brasil, São Paulo, Brazil).

The chemical conditions of the cultivated soil were assessed by collecting ten subsamples from the 0–30 cm soil layer in the experimental area; these samples were then combined to form a representative composite sample. The results of the analysis are shown in Table 2.

### 2.4. Spectral Data Acquisition

The spectral readings obtained with the AS7265x optical sensor and the SPAD-502 (Konica Minolta, Tokyo, Japan) chlorophyll meter were standardised for the last newly formed leaf of one plant per experimental plot. The SPAD-502, a widely used tool for nitrogen management in precision agriculture, determines leaf chlorophyll content by measuring light absorption at two specific wavelengths, 650 nanometres for red light and 940 nanometres for infrared radiation. This measurement serves as an indirect indicator of leaf nitrogen concentration and has been successfully applied in studies evaluating nitrogen fertilisation strategies. In particular, Martins et al. [20] employed the SPAD-502 to assess nitrogen sufficiency and optimize fertilizer application rates in corn crops using a variable-rate approach, highlighting the device’s role in improving nitrogen use efficiency while maintaining crop productivity. The area for the spectral readings was standardised to obtain the average of three points on the middle third of the adaxial face of the leaf blade. The samples from each experimental unit were analysed at phenological stages V5, V10, and R2 (13, 27, and 53 days after planting, respectively), resulting in 72 leaf-tissue samples collected throughout the experiment. After acquisition of the in situ data, the samples were removed, identified, stored in paper bags, placed in a Styrofoam box, and immediately taken to the laboratory. Each sample was analysed using a FieldSpec PRO FR 3 spectroradiometer (Analytical Spectral Devices Inc., Boulder, CO, USA), a hyperspectral instrument designed for acquiring high-precision reflectance measurements across the visible-to-near-infrared (VNIR) and short-wave infrared (SWIR) spectral ranges. The FieldSpec operates within a spectral range of 350–2500 nm, ensuring comprehensive spectral characterisation of plant tissues. Continuous spectral readings of the samples were acquired in a darkroom under climate-controlled conditions, employing a Hi-Brite Contact Probe in combination with the spectroradiometer. This non-imaging sensor integrates three spectrometers with spectral resolutions of 3 nm and 10 nm (resampled to 1 nm). The spectral measurements were carried out continuously in an air-conditioned dark room, using the Hi-Brite Contact Probe. The FieldSpec was calibrated for reflectance every 15 min, with a standard reading taken on a Spectralon plate. The Digital Number (DN) values of the samples were converted into a reflectance factor using ViewSpecPro 6.2 software.

### 2.5. Laboratory Analysis

After being analysed with the FieldSpec, the samples were pre-dried in an oven at 60 °C for 48 h or until reaching constant weight. After undergoing pre-drying, the samples were macerated using a mortar, placed in duly identified transparent plastic bags (5 × 24 cm), and stored in the dark under low-humidity conditions. The samples, weighing 0.1 g each, were then taken to the laboratory. The leaf nitrogen concentrations (LNCs) of the samples were determined using the method proposed by Kjeldahl [21] in g of N per kg of leaf-tissue dry matter.

### 2.6. Data Processing and Statistical Analysis

LNC data from the samples were subjected to the Kolmogorov–Smirnov test of normality using the ‘stats’ statistical package of the software product R 4.5 [22]. The correlation coefficient (r) was then evaluated to relate potential spectroradiometer bands to the behaviour of the LNCs. The ‘hsdar’ package was used in RStudio 2023.12.1 [23] to verify the NRI (normalised ratio indices) (Equation (1)) across all bands of the optical instruments for correlation with the LNCs. The linear regression of each NRI with the LNC data was represented by graphs of the coefficient of determination (R^2^) between the analysed bands. The indices that afforded the best R^2^ values, according to phenological stage, were then selected for later cross-validation via the k-fold method (k = 4), using the ‘caret’ package [24] in RStudio. The use of 4-fold cross-validation is justified by the need to balance the available data, ensuring a structured division between training and validation samples in each iteration. This approach helps provide a reliable assessment of model performance, especially when working with a dataset of limited size.

(1) $${NRI}_{\left(B1,B2\right)}=\frac{\rho_{B1}-\rho_{B2}}{\rho_{B1}+\rho_{B2}}$$

**Figure 3:**
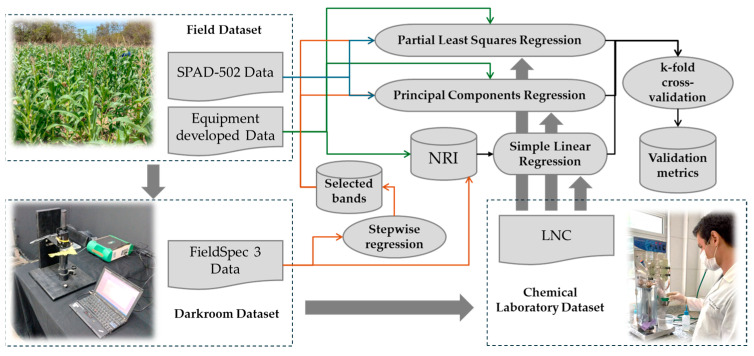
Flowchart of the Statistical Data Analysis Process.

In a second approach, Partial Least Squares Regression (PLSR) and Principal Component Regression (PCR) were used to develop predictive models for the LNC using data from FieldSpec 3 spectroradiometer; the experimental device; and the SPAD-502. These algorithms were chosen for their ability to handle the high dimensionality and collinearity of spectral data. PLSR projects latent variables that optimise the covariance between predictors and the response variable, and PCR isolates principal components from the predictors for subsequent regression, reducing dimensionality and enhancing model interpretability. Due to the volume of spectral data per instrument, the best bands were selected via Stepwise regression using the ‘stepAIC’ function of the ‘MASS’ package in R [25], in which the Akaike Information Criterion (AIC) is applied as the selection criterion in a bidirectional stepwise procedure. After developing the models, k-fold cross-validation was carried out with k = 4. Following validation, the models were evaluated using the Mean Absolute Error (MAE), Root Mean Square Error (RMSE), and Coefficient of Determination (R^2^). These methodological enhancements improved both the accuracy and robustness of the predictive models. Figure 3 presents a flowchart depicting the analyses performed with the datasets acquired from the field, the darkroom, and the LNC determination.

## 3. Results

### 3.1. Device Assembly and Calibration

The construction process resulted in a new portable optical device, the MSPAT (Multispectral Soil Plant Analysis Tools). The features incorporated into the device included data acquisition and transmission via Wi-Fi, graphic communication via an OLED display, and the ability to store spectral data together with geolocation information.

**Figure 4:**
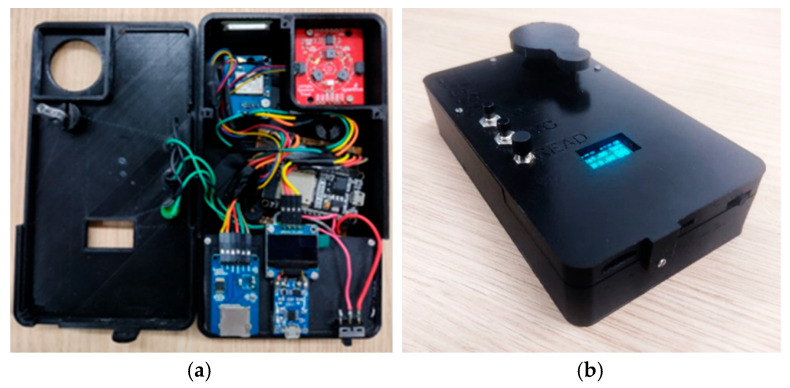

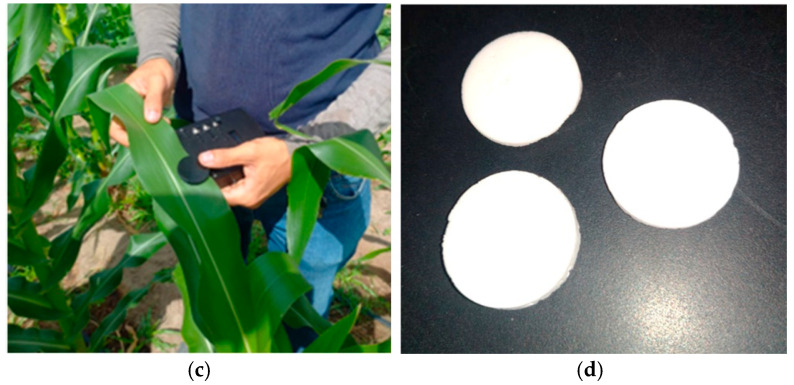
Assembly and operation of the MSPAT: (**a**) electronic wiring, (**b**) device in operation, (**c**) field evaluation using maize, and (**d**) calibration plate made of sintered barium sulphate.

**Figure 5:**
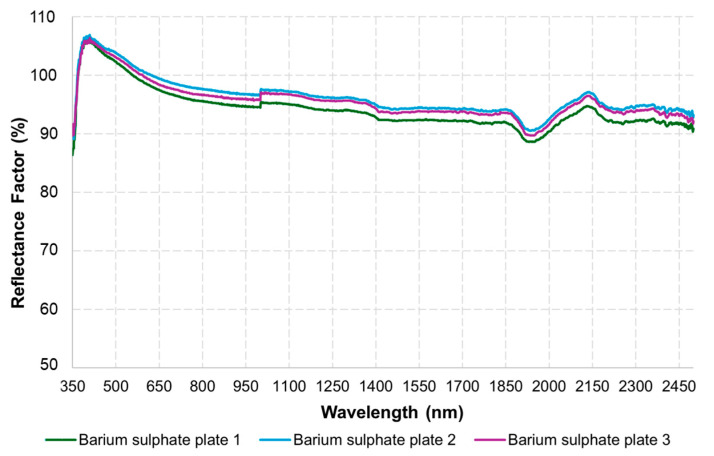
Spectral behaviour of the barium sulphate calibration plate.

Figure 4a shows the interior of the MSPAT after all the electronic components were connected, while Figure 4b shows the device in operation for data acquisition. Figure 4c shows the MSPAT being used to evaluate maize crops in the field experiment. The sintered barium sulphate (Figure 4d) produced a highly reflective white plate with relevant optical properties (Figure 5), measured in terms of the reflectance factor calibrated with a Spectralon plate. Due to its high reflectance and diffuse light-scattering characteristics, this material ensures reliability and accuracy in optical sensor calibration. Additionally, it exhibits uniform reflectance behaviour across the wavelengths used by the AS7265x sensor, a characteristic that is essential for maintaining consistency in spectral measurements. Its suitability as a reference standard is particularly valuable for applications requiring precision and consistency, reinforcing the methodology established by Poh et al. [19].

### 3.2. Statistical Analysis

**Figure 6:**
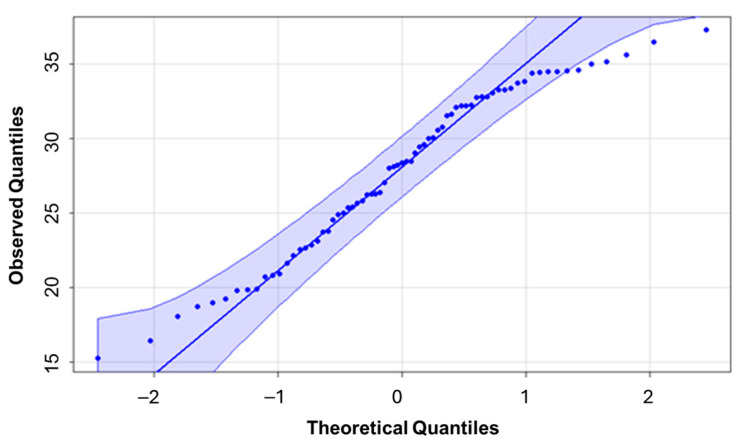
Test of normality for the leaf nitrogen concentration data using Kolmogorov–Smirnov statistics.

The LNC data showed a normal distribution (Figure 6), according to the Kolmogorov–Smirnov test (*p*-value 0.2884). The data therefore meet the assumption of normality required for the application of parametric statistical analyses and the development of predictive models.

**Figure 7:**
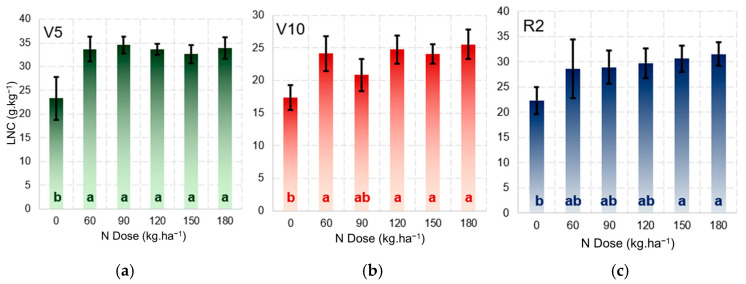
Mean leaf nitrogen concentration at phenological stages V5 (**a**), V10 (**b**), and R2 (**c**) for different nitrogen treatments. The letters inside the bars indicate statistical differences between treatments; different letters represent significant differences according to Tukey’s test (*p* ≤ 0.05).

The ANOVA revealed significant differences (*p* < 0.05) in the LNCs at stages V5, V10, and R2 for the N treatments applied. Tukey’s test showed the statistical difference between the control (N0) and the other treatments at the three phenological stages (Figure 7), demonstrating that starting from 30 kg·ha^−1^ N (Table 1), the treatments produce different LNC values. The treatments at stages V10 and R2 were mainly influenced by the fertilisations carried out 14 and 42 days after planting, with the N150 treatment being the optimal dose for maintaining the LNC in maize under the conditions of the experiment. A range of different field conditions were provided in the experiment to the express different LNC responses in the maize.

### 3.3. Spectral Analysis

**Figure 8:**
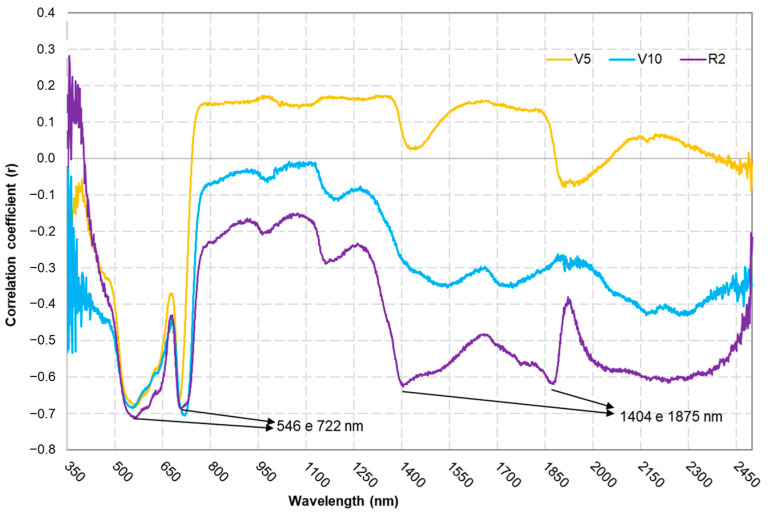
Correlation coefficients (r) between the spectroradiometer bands and leaf nitrogen concentrations at different phenological stages.

The correlation coefficient (Figure 8) between the spectroradiometer bands and the LNC showed an increased correlation in the near-infrared (NIR) region throughout each phenological stage. Specifically, the 546 nm (green) band exhibited a strong negative correlation of −0.7102, and that for the 722 nm (red edge) band reached −0.704, whereas the 1404 nm and 1875 nm bands displayed lower negative correlations of −0.6243 and −0.6187, respectively, indicating that not all the coefficients uniformly approached −0.7.

**Figure 9:**
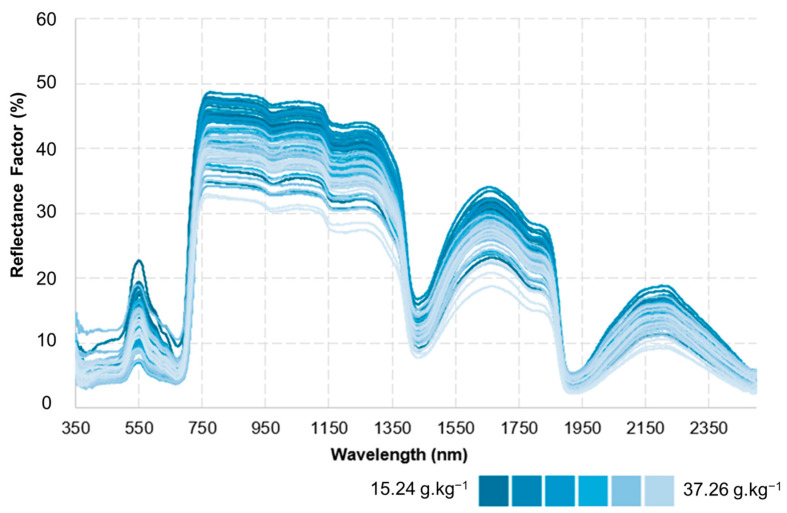
Spectral Response of Maize Leaves as a Function of Nitrogen Concentration.

Figure 9 illustrates the spectral behaviour of leaves as a function of nitrogen concentration. The blue-tone scale used to represent reflectance values shows that leaves with lower nitrogen concentrations exhibit higher reflectance, identified by darker blue shades. Conversely, leaves with higher nitrogen concentrations display lower reflectance values, highlighted by lighter blue tones. These results confirm the inverse relationship between foliar nitrogen content and spectral reflectance, demonstrating the influence of a leaf’s chemical composition on its spectral response.

**Figure 10:**
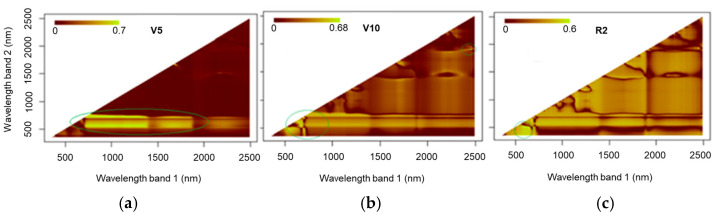
Coefficient of determination (R^2^) of normalised ratio indices (NRIs) calculated using spectroradiometer data at phenological stages V5 (**a**), V10 (**b**), and R2 (**c**).

Figure 10 shows the values for R^2^ in relation to the NRIs for the models at stages V5, V10, and R2 obtained using data acquired via Fieldspec 3. At stage V5, more relevant results (R^2^= 0.7) were observed when using bands from the green to red-edge region (500 to 750 nm) and the NIR range (700 to 1400 nm) to generate NRI indices. At stage V10, high values for the coefficient of determination (R^2^ = 0.69) were observed in the region of the green spectrum (480 to 550 nm) combined with the red bands (610 to 670 nm) as well as with the SWIR (1500 to 2500 nm). In stage R2, there was a significant contribution from the infrared bands; however, the most significant values for the coefficient of determination (R^2^ = 0.6) were seen when using bands in the visible spectrum.

**Figure 11:**
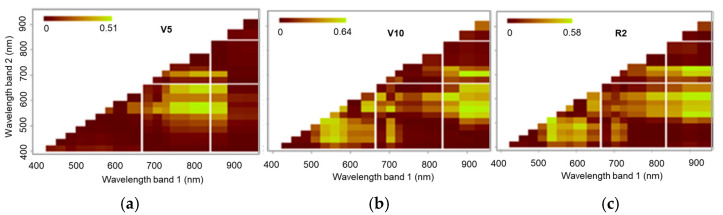
Coefficient of determination (R^2^) of normalised ratio indices (NRIs) calculated using MSPAT data at phenological stages V5 (**a**), V10 (**b**), and R2 (**c**).

The results for the NRIs using the MSPAT data (Figure 11) show that at stage V5, the 585 nm band, as a normalised ratio with the 810 and 860 nm bands, afforded the best correlations with the LNC. At stages V10 and R2, similar results were found when the 900 and 940 nm bands were used together with the 585 and 610 nm bands. At stages V10 and R, there was also a contribution from indices between the visible bands but with lower values for the coefficient of determination (R^2^).

**Figure 12:**
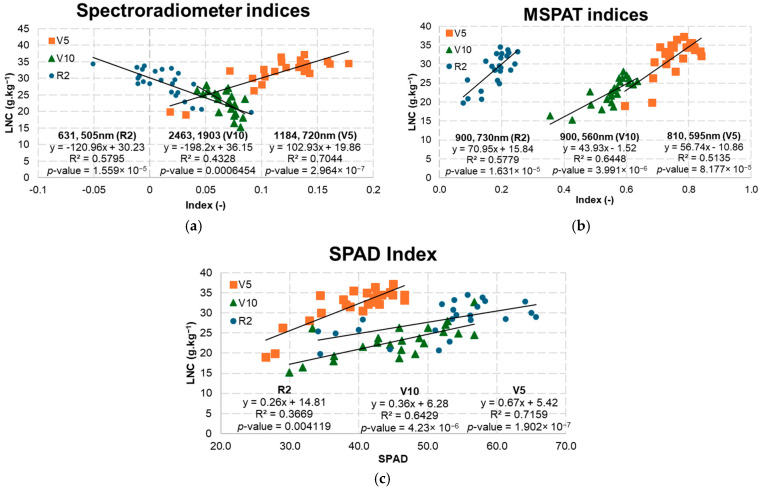
Models selected by optical instrument and phenological stage using the spectroradiometer (**a**), MSPAT (**b**), and SPAD (**c**).

The best NRIs obtained with the spectroradiometer (Figure 12a) were acquired using the NIR (1184 nm) as a normalised ratio with the red-edge band (720 nm) at stage V5, resulting in an R^2^ of 0.7044. The other indices with the highest values for R^2^ were obtained using the visible bands (631 and 505 nm) at stage R2 and bands between the SWIR (2463 and 1903 nm) at stage V10. In relation to the MSPAT (Figure 12b), using the NIR bands as a normalised ratio with the red-edge (730 nm) or green (560 and 595 nm) bands gave a better fit for the model at stage V10, with an R^2^ of 0.6448. The results obtained by using the SPAD index (Figure 12c) to estimate the LNC at phenological stage V5 were significant (R^2^ = 0.7159). However, as the crop developed, R^2^ values declined to 0.71, 0.50, and 0.36 (Figure 12), accompanied by a reduction in the angular coefficients of the models (0.67, 0.37, and 0.28).

### 3.4. Predictive Modelling and Validation

**Figure 13:**
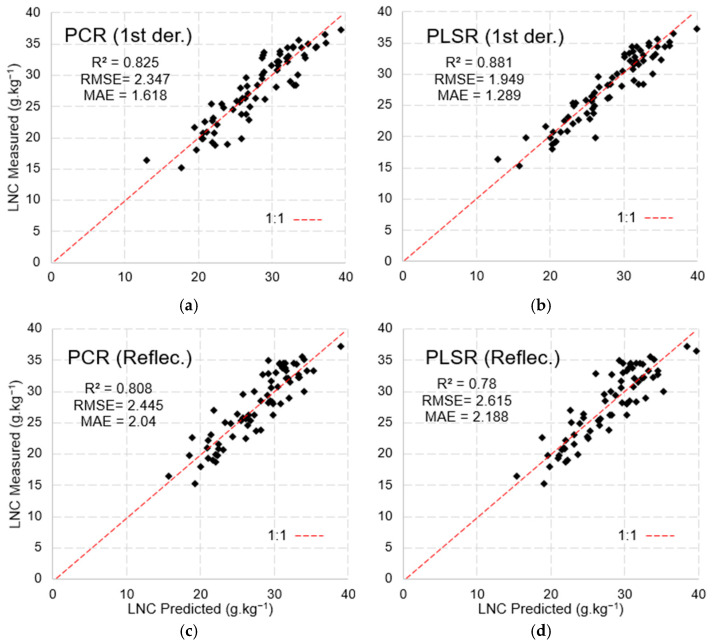
Model validation using spectroradiometer data: (**a**) first derivative of the PCR model, (**b**) first derivative of the PLSR model, (**c**) raw PCR data, and (**d**) raw PLSR data.

The models in Figure 13 were evaluated using the validation metrics shown in Table 3, from which it was found that the indices applied to stages V5 and V10 by the optical instruments were more accurate in predicting the LNC. At stage V10, the MSPAT showed better predictive ability, reaching an RMSE of 2.257, while the RMSE for SPAD reached 2.253. For the spectroradiometer, the best result was seen at stage V5, with an RMSE of 2.036.

Table 4 shows the bands selected via the stepwise method applied to the PLSR and Principal Component factors, using data obtained with the spectroradiometer and MSPAT, which are sensitive to leaf nitrogen concentration (LNC). The PLSR approach not only reduced the number of factors but also yielded the best coefficient of determination when calibrating the models (R^2^ = 0.882). This model, based on the data from the spectroradiometer with the first-order derivative, stands out from the others due to the significant contribution of the bands in the SWIR (1675 to 2374 nm). For the models using reflectance data, verifying the weight of the parameters showed that the green band (557 and 561 nm) from FieldSpec 3 and red band (645 nm) from the MSPAT were more relevant when estimating the LNC.

The k-fold cross-validation applied to the PCR and PLSR models using data from the spectroradiometer (Figure 13) reinforces the effectiveness of the models in predicting LNCs. In the case of PCR (Figure 13a,c), there were slight variations in the error metrics, with the first-derivative data exhibiting the best performance (R^2^ = 0.82, RMSE = 2.35, MAE = 1.62). The PLSR model (Figure 13b), when using first-derivative reflectance data, generated more robust validation metrics (R^2^ = 0.88, RMSE = 1.95, MAE = 1.29) than the PCR model.

**Figure 14:**
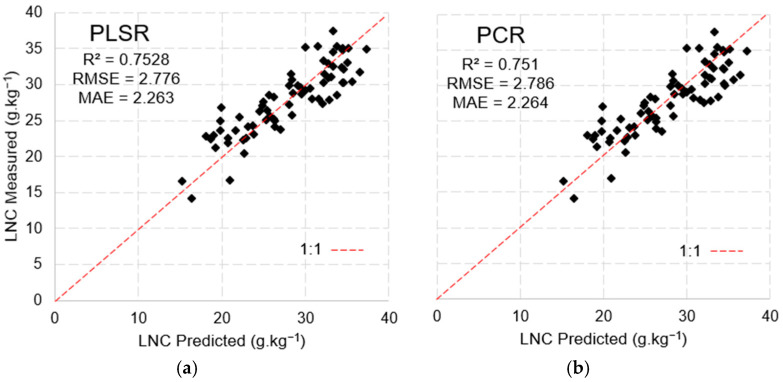
Model validation via PLSR (**a**) and PCR (**b**) with MSPAT data using the k-fold validation method.

The model developed using PLSR with the MSPAT data explained 70.8% of the variance with six factors, while PCR explained 68% of the variance, including nine factors. The results of the validation (Figure 14) show the remarkable ability of both methods in predicting the LNC; however, PLSR yielded the better performance (R^2^ = 0.712, RMSE = 2.99 and MAE = 2.43).

## 4. Discussion

The MSPAT’s development involved incorporating electronics, programming, and 3D printing to enable the acquisition of spectral data in agricultural areas, allowing the application of reflectance data to targets in different regions of the spectrum. Given the potential of the AS7265x sensor, recent studies have also demonstrated its successful use in detecting the ripeness of apples in the field [26], estimating the moisture content of maize grain [27], and monitoring photosynthetically active radiation (PAR) [8]. The barium sulphate plate (Figure 4d) manufactured to calibrate the sensor was a crucial element in regard to the quality of the data obtained by the device. However, it should be noted that in certain contexts, the Spectralon^®^ plate offers superior results in terms of reflectance [19], as shown in Figure 5. The advantages of developing the barium sulphate plate used in this study, including its design specifically for the MSPAT, are the high accessibility of the materials required for its development and the simplicity of the manufacturing process.

In Figure 7, the control shows a significant difference in relation to the other treatments throughout each of the phenological stages due to the high rate of development and assimilation of N, especially during the vegetative phase [28]. Stages V10 and R2 show some depletion in the levels of soil N, a result of the average LNC in the treatments with 60, 90, and 120 kg·ha^−1^ N (Figure 7b,c). The relationship between nitrogen fertilisation and the LNC has been the subject of various studies [29,30,31]; however, the present study highlights and validates the effect on maize under the environmental conditions used for demonstrating the representative models. 

Yoder and Pettigrew-Crosby [32] also identified a strong correlation between the 550 and 730 nm bands and LNCs. These results show that the green and red-edge regions have great potential for use in measuring LNC due to the influence of the cellular structure of the leaves and the chlorophyll concentration [33]. Furthermore, when analysing the r value during each phenological stage (Figure 8), an increase can be seen in the influence of the infrared wavelength, especially in the water absorption bands (1404 and 1875 nm), corroborating the findings of Chi et al. [34], which show that water content is a limiting factor for the LNC during the reproductive stage.

The spectral patterns shown in Figure 9 indicate that leaves with higher nitrogen concentrations exhibit greater absorption of electromagnetic radiation, particularly in the red and near-infrared (NIR) regions, due to the presence of compounds such as chlorophyll and proteins. Consequently, the lower reflectance detected in leaves with high nitrogen levels reflects this more intense absorption. On the other hand, leaves with lower nitrogen content show higher reflectance, which may be associated with reduced cellular density and lower photosynthetic activity, leading to decreased absorption of incident radiation.

The results for the NRIs (Figure 10) show that, in general, the NIR bands contributed to the indices that yielded the highest R^2^ values. This is due to the health of the cellular structure and the nitrogen concentration of the plants being associated with this region of the spectrum [35]. For the NRIs generated using data from the spectroradiometer, the best results were seen at stage V10, where the SWIR bands stood out (R^2^ = 0.704), providing a superior fit compared to the other indices. However, after validation (Table 3), the model showed low replication potential (R^2^ = 0.587). Going beyond the traditional relationship between VNIR (visible and near-infrared) bands and leaf pigments, this study is in agreement with Berger et al. [13], who suggested the use of SWIR bands to estimate nitrogen concentrations, highlighting the strong relationship between this spectral region and leaf proteins.

Uchino et al. [36] used the SPAD-502 to estimate LNCs and found that during the vegetative stage, the regression models obtained a fit to the coefficient of determination that ranged from 0.78 to 0.88, while during the reproductive stage, the fit ranged from 0.27 to 0.54. These results corroborate those of the present study (Figure 12c). The reduction in the predictive capacity for the LNC during the cycle is mainly due to the onset of the reproductive phase, as the reproductive organs become the primary sources of energy expenditure for the plant [37] and, consequently, assimilated nitrogen. It is therefore understood that, due to physiological changes in a plant, it may not be possible to apply the developed models to other phenological stages.

The MSPAT integrated with the NRIs used for predicting LNC yielded results similar to those produced by the SPAD index (Table 3). This may be related to the limitations of the indices in terms of using only two bands to explain the behaviour of LNCs. When we applied the multivariate statistical approach using PLSR and PCR, the MSPAT proved to be a better tool than SPAD-502 for estimating the LNC based on the validation parameters shown in Table 3 and Figure 13. It should also be remembered that the technical and spectral characteristics of the device may favour different results, since while SPAD-502 analyses the transmittance of the leaf tissue, the MSPAT sensor measures reflectance.

The validation results for the predictive models using PLSR and PCR (Table 4) are similar to those found by Zhang et al. [38], who evaluated different levels of N in the leaves of cotton plants and found an R^2^ of between 0.80 and 0.84 using first-derivative PCR data, while the PLSR models gave an R^2^ of 0.79 to 0.81. Their study also evaluated the performance of the models using raw reflectance data and found that, unlike in the present study, there was no difference between the two approaches. PLSR is a well-established method in spectroradiometry and chemometrics for developing predictive models, as it effectively reduces multicollinearity and maximizes the covariance between predictor and response variables. As such, it differs from PCR, as it reduces the dimensionality of data by extracting the principal components without considering their relationship with the dependent variable, whereas PLSR simultaneously models independent variables and the dependent variable, ensuring that the extracted components are relevant to the prediction [39]. Meacham-Hensold et al. [40] examined models for predicting LNCs in tobacco leaves using PLSR and obtained an R^2^ of 0.81. The models in the present study showed similar performance, achieving an R^2^ of 0.78 using raw reflectance data measured with a spectroradiometer.

## 5. Conclusions

This study demonstrates the potential of the MSPAT as an effective tool for estimating LNCs in maize, contributing to the optimisation of nitrogen fertilisation and the advancement of precision agriculture. The findings confirm that the MSPAT provides reliable accuracy in spectral analysis, achieving validation results comparable to those obtained with FieldSpec 3 and outperforming SPAD-502. The NRIs generated from the spectral data demonstrated strong correlations, with the most effective index utilising the 1184 and 720 nm bands. Additionally, multivariate analysis highlighted the robustness of PLSR when applied to first-derivative spectral data, reinforcing the device’s capacity for precise nitrogen concentration assessment.

Future studies could refine nitrogen management strategies to enhance fertilisation efficiency and optimise the use of spectral indices for different phenological stages. Improvements in the sensor’s calibration and integration with machine learning models could further strengthen its predictive capacity. Moreover, combining the MSPAT with remote sensing technologies may enable multi-scale agricultural monitoring, expanding this device’s applicability in regard to large-scale precision farming systems. The integration of the MSPAT into precision agriculture frameworks has the potential to revolutionise nitrogen application practices, ensuring greater resource efficiency and environmental sustainability.

## Figures and Tables

**Table 1:** Plan of nitrogen fertilisation for the experiment.

Days After Planting (DAP)	Experimental Treatments					
	N0	N60	N90	N120	N150	N180
0	0	30	30	30	30	30
14	0	30	60	60	90	90
28	0	0	0	30	30	30
42	0	0	0	0	0	30
Total	0	60	90	120	150	180

**Table 2:** Chemical characteristics of the soil in the experimental area.

Assortative Complex (cmol_c_/kg)										
Ca^2+^	Mg^2+^	Na^+^	K^+^	H^+^ + Al^3+^	Al^3+^	S	T	V (%)	m (%)	PST
1.55	1.03	0.22	0.19	0.66	0	3	3.6	82	0	6
C (g/kg)	N (g/kg)	C/N	OM (g/kg)	P (mg/kg)		pH (Water)	EC (dS/m)			
5.26	0.54	10	9.07	9		6.5	0.07			

**Table 3:** Validation metrics for predictive models of leaf nitrogen concentration (LNC) across instruments and phenological stages.

	FieldSpec 3				SPAD			MSPAT	
Stage	R^2^	RMSE	MAE	R^2^	RMSE	MAE	R^2^	RMSE	MAE
V5	0.7890	2.0361	1.6719	0.6710	2.5660	2.0629	0.6107	3.4144	3.0172
V10	0.5870	2.5874	2.1179	0.7091	2.2530	1.7375	0.7935	2.2577	1.8279
R2	0.6845	2.8562	2.6229	0.4231	3.6987	3.0395	0.6855	2.8664	2.4704

**Table 4:** Wavelengths selected for predicting the leaf nitrogen concentrations using the PCR and PLSR models with the spectroradiometer and MSPAT data.

	FieldSpec 3 (Reflectance)			FieldSpec 3 (First Derivative)			MSPAT (Reflectance)		
	Bands	PCR	PLSR	Bands	PCR	PLSR	Bands	PCR	PLSR
Model parameters	364 nm	−246.11	−310.61	543 nm	−14,642.64	−18,476.88	410 nm	133.35	132.47
	369 nm	−636.74	−655.29	657 nm	19,950.06	9443.36	460 nm	−62.01	−65.21
	374 nm	430.83	501.34	660 nm	−14,339.63	−6140.98	485 nm	134.57	130.40
	388 nm	−412.56	−328.38	995 nm	−15,891.36	−20,518.20	535 nm	−133.07	−124.83
	397 nm	1015.84	931.40	1675 nm	−23,383.25	−20,468.88	560 nm	−51.19	−86.50
	557 nm	1611.30	−18.74	2155 nm	−9291.63	−3923.69	585 nm	−56.85	−21.36
	561 nm	−1727.78	−78.68	2189 nm	−15,009.74	−14,560.58	610 nm	48.27	48.72
	735 nm	−87.52	−67.73	2257 nm	22,502.14	22,948.61	645 nm	−193.43	−197.96
				2279 nm	−17,066.27	−19,334.37	680 nm	−126.33	−124.62
				2283 nm	−10,996.14	−9975.65	705 nm	124.30	133.04
				2374 nm	5773.32	5767.96	730 nm	−61.85	−62.01
							810 nm	54.23	53.91
Intercept		61.96	59.74		40.80	41.57		42.95	42.29
R^2^		0.808	0.781		0.826	0.882		0.751	0.753
Number of factors		8	6		10	6		11	9

## Data Availability

Dataset available on request from the authors.
